# High Channel Temperature Mapping Electronics in a Thin, Soft, Wireless Format for Non-Invasive Body Thermal Analysis

**DOI:** 10.3390/bios11110435

**Published:** 2021-11-02

**Authors:** Wooyoung Park, Chunki Yiu, Yiming Liu, Tsz Hung Wong, Xingcan Huang, Jingkun Zhou, Jian Li, Kuanming Yao, Ya Huang, Hu Li, Jiyu Li, Yanli Jiao, Rui Shi, Xinge Yu

**Affiliations:** 1 Department of Biomedical Engineering, City University of Hong Kong, Hong Kong 999077, China; wypark2@um.cityu.edu.hk (W.P.); chunkiyiu2-c@my.cityu.edu.hk (C.Y.); lyiming2-c@my.cityu.edu.hk (Y.L.); thwong247-c@my.cityu.edu.hk (T.H.W.); xhuang439-c@my.cityu.edu.hk (X.H.); jingkzhou3-c@my.cityu.edu.hk (J.Z.); jian.li@my.cityu.edu.hk (J.L.); km.Yao@my.cityu.edu.hk (K.Y.); yhuang@hkcoche.org (Y.H.); huli23@cityu.edu.hk (H.L.); jiyuli2-c@my.cityu.edu.hk (J.L.); yanlijiao2-c@my.cityu.edu.hk (Y.J.); rshi@connect.hku.hk (R.S.); 2 Hong Kong Center for Cerebra-Cardiovascular Health Engineering, Hong Kong Science Park, New Territories, Hong Kong 999077, China

**Keywords:** skin-like electronics, wireless communication, human temperature measuring, flexible electronics, thermistor

## Abstract

Hemodynamic status has been perceived as an important diagnostic value as fundamental physiological health conditions, including decisive signs of fatal diseases like arteriosclerosis, can be diagnosed by monitoring it. Currently, the conventional hemodynamic monitoring methods highly rely on imaging techniques requiring inconveniently large numbers of operation procedures and equipment for mapping and with a high risk of radiation exposure. Herein, an ultra-thin, noninvasive, and flexible electronic skin (e-skin) hemodynamic monitoring system based on the thermal properties of blood vessels underneath the epidermis that can be portably attached to the skin for operation is introduced. Through a series of thermal sensors, the temperatures of each subsection of the arrayed sensors are observed in real-time, and the measurements are transmitted and displayed on the screen of an external device wirelessly through a Bluetooth module using a graphical user interface (GUI). The degrees of the thermal property of subsections are indicated with a spectrum of colors that specify the hemodynamic status of the target vessel. In addition, as the sensors are installed on a soft substrate, they can operate under twisting and bending without any malfunction. These characteristics of e-skin sensors exhibit great potential in wearable and portable diagnostics including point-of-care (POC) devices.

## 1. Introduction

Healthcare monitoring electronic devices which measure various clinically relevant diagnostic parameters (e.g., electrocardiogram, blood pressure, and respiratory rate) have been considered as one of the fundamental medical apparatus for evaluating the health status of patients before establishing a detailed therapeutic scheme [1,2,3,4,5,6,7,8,9,10]. Among the diagnostic variables, values elaborating vital signs of the vascular system including thermal information, pressure, pulse, blood flow, blood composition, and oximetry are deemed significant as these measurements can indicate the presence of physiological diseases such as diabetes, arteriosclerosis, and angiosarcoma [11,12,13,14,15,16,17]. Up to now, medical imaging technologies adopting ionizing radiation or radio waves (e.g., angiography, computed tomography (CT), magnetic resonance imaging (MRI), and laser Doppler flowmetry) have been the most commonly employed technologies for analyzing the blood circulatory system [18,19,20,21]. However, although the aforementioned techniques have proven their high accuracy and performance, these hardly meet the requirement of daily health care and emergency treatment due to their bulky physical size and complicated operation procedures [22,23].

Medical imaging technologies have been developed as significant analyzing tools in the medical field since the innovative invention and use of X-ray in the late 19th century [24]. As the majority of modalities exploit ionizing radiation as a detecting source for the internal body structures (e.g., organs, tissues, and bones) [25,26,27,28], apparent and accurate images of the targeted segment are obtained according to its programmed dose rate [29,30]. Despite their notable performance, the conventional imaging techniques have crucial flaws, including (1) risks of radiation exposure that is a potential cause of cancer [26,31,32], (2) lengthy planning procedures and operating duration which are inadequate for emergencies, (3) high cost, and (4) bolus injection of contrast media or magnetic microspheres that possibly induces allergic reactions and even, failure in specific organs [33,34,35]. Consequently, wearable and flexible biosensors that exploit soft electronics have been suggested as the corresponding solution for health monitoring on account of their notable physical and mechanical properties including an ultra-thin thickness, light-weight, and high strain ratio [36,37,38,39,40,41,42,43,44,45,46].

With the rapid development of flexible electronics, a wide range of noticeable applications have been built in the biomedical field [47,48,49,50,51,52,53,54,55,56,57] as noninvasive point-of-care (POC) devices to monitor human health status in real-time. Especially for hemodynamic status exhibiting the overall physiological condition, the usage of wearable electronics has drawn significant attention due to their availability for noninvasive [58], wireless, safe, and instantaneous measurement by simple wearing in contrast to the conventional imaging technologies [59,60]. Currently, most modalities of wearable hemodynamic monitoring sensors exploit mechanical variables (e.g., pressure and strain) as their key measuring factors [61,62,63]. However, limitations still exist, including (1) low reliability resulting from the characteristics of mechanical sensors (nonlinearity and large hysteresis) [64], (2) a lack of stability derived by fatigue from cyclic motions [65,66], (3) relatively high cost due to complicated manufacturing procedures [67], and (4) a limited number of target diagnostic values (e.g., blood pressure and heartbeat rate) resulting from a single physiological parameter (blood pulse) [63,68,69]. Thus, wearable temperature mapping technology has been highlighted as an alternative due to its competitive characteristic strengths over the aforementioned sensors. Specifically, through blood vessel thermal analysis, vascular disorders such as hypertension, hypoperfusion, and arteriosclerosis can be detected according to the temperature gradients of the target circulatory system [12,70,71,72]. At the same time, the thermal sensors illustrate significant sensitivity enabling exquisite detection of temperature change [73,74]. Furthermore, compared to the mechanical sensors, thermal sensors have higher durability as they are not repeatedly exposed to fatigue during operation [75]. Therefore, wearable thermal analysis technology has been emerging as the next generation of hemodynamic monitoring devices.

Herein, we report a wearable thermal analysis system in a thin, soft, and flexible format, named as a wearable hemodynamic sensor (WH-sensor), that allows for the hemodynamic status of specific blood vessels to be monitored effectively and conveniently in a wearable and non-invasive way. A high-density sensing array associates with 80 thermal sensing units based on negative temperature coefficient (NTC) thermistors with remarkable sensitivity, serving as the key components for thermal analysis, which integrates on a soft substrate to segment the detecting region. The data transmitted from sensing channels are operated through multiplexers, electrical components increasing the number of channels. As the surface-mount device (SMD) type of thermistors with a size of 1.0 mm × 0.5 mm (length × width) are adopted, the resolution of the monitoring system is improved while the extent of the device is maintained tight. Furthermore, for real-time monitoring, a graphical user interface (GUI) is implemented wirelessly via Bluetooth modules on the screen of external electronic devices such as a computer and smartphone. Through this GUI software, the blood vessel and hemodynamic status can be detected and evaluated in colors on the GUI indicating the level of heat according to the sensed temperature. The equipment that is directly attached to a skin senses a greater heat for the region where a blood vessel is. Even on the condition that there is a blockage due to the presence of thrombus and cholesterol on the wall of the vessel, a reduced diameter of the blood pathway would induce noticeably less heat because of a decrease in the volumetric flow rate of the blood according to the fluid dynamic principle [76,77,78]. Therefore, by simply wearing this twistable and bendable device, the location of blood vessels can be detected accurately and conveniently for medical purposes (e.g., bolus injection and venipuncture) [79,80,81]. At the same time, through hemodynamic analysis, conditions like atherosclerosis which is one of the main causes of fatal vascular system diseases (e.g., stroke, heart attack, and blood vessel rupture) could be prevented in advance [11,82,83]. With the development of skin-integrated sensors, it is feasible to benefit users immensely by providing them POC devices with wearability, light-weight, and convenient measurements [84,85].

## 2. Results and Discussion

**Figure 1:**
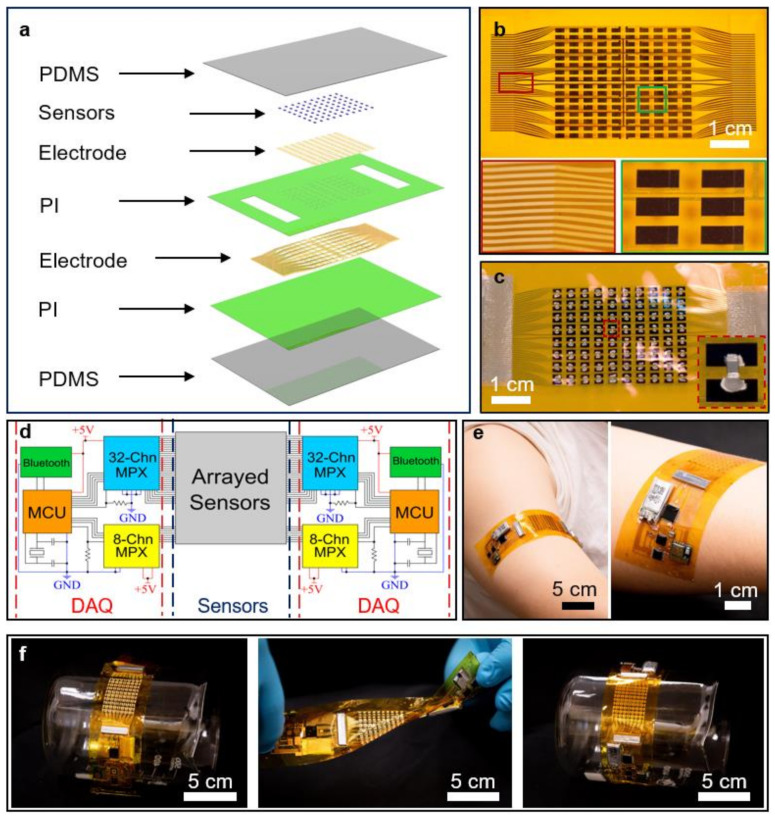
Overview of the WH-sensor. (**a**) Schematic illustration of the sensing panel of the WH-sensor. (**b**) Optical images of the sensing panel of the device with enlarged regions of conductive patterns. (**c**) Optical images of the sensing panel of the device with enlarged regions of soldered sensors. (**d**) Equivalent circuit diagram of the WH-sensor. (**e**) Optical images of the device attached to the right arm of a volunteer during operation. (**f**) Optical images of the device under bending inward, twisting, and bending outward.

Figure 1a shows the schematic diagram of the thermal sensing panel of the WH-sensor. From the bottom to the top, the structural design of the sensing unit of the device consisting of a layer of polydimethylsiloxane (PDMS), two thin polyimide (PI) films where a lower conductive electrode is sealed in between, an upper electrode for sensors, thermal sensors, and another layer of PDMS is clearly shown. The lower PI layer serves as a supporting layer for the lower metallic electrode (Au) that is a conductive pattern to manage the electrical power over the whole circuit in the sensing panel of the device. Similarly, another conductive electrode (Au) for connecting the arrays of thermal sensors (thermistors) to the lower electrode was deposited on the upper PI layer. Particularly, the top PI film is with holes and two rectangular gaps at the ends to link the two layers of electrode and the sensors to external devices for measurement respectively. In the device, the surface-mount device (SMD) type of a negative temperature coefficient (NTC) thermistor (NTCG103JX103DT1S) was employed due to its compact size (1.0 mm × 0.5 mm (length × width)). Consequently, according to the electrical characteristics of the NTC thermistor, its resistance is inversely proportional to the applied temperature. Finally, two PDMS layers at the bottom and top encapsulated the layers of the sensing system for protection. Figure 1b,c present the entire sensing panel of the WH-sensor before and after the installation of electrical components (thermistors and anisotropic conductive film (ACF)) with enlarged conductive patterns. The thermal resistors were attached to the upper electrode by applying a silver paste as temperature sensors while the AFCs containing particles with conductivity were utilized to provide electrical pathways for measuring devices such as a voltage meter. Therefore, variable resistances generated by the sensors according to their stimuli could be observed via a voltmeter. In total, 80 thermistors in the array of 8 × 10 (row × column) were mounted on the sensing panel with a total size of 7.8 cm × 3.8 cm × 0.17 mm (length × width × thickness) to perform as NTC thermal sensors. As a result, the temperatures of 80 subdivided regions on the target area could be measured simultaneously. Furthermore, to increase the resolution of the temperature mapping system, the gaps among each thermistor in the array were minimized by adopting a straight-line design of the conductive layer in the sensing panel [86,87]. According to the data collected, the location of a specific blood vessel and its flow direction can be analyzed at the same time. For operating 80 thermal sensors concurrently, two controlling panels that manage 40 channels of sensors were merged with one sensing panel of the device. For each controlling unit operated by a commercial Lithium-ion battery (4.2 V), a microcontroller unit (MCU), Atmega328P, was applied for processing the sensing data from 40 thermal sensors of the WH-sensor while two different types of multiplexers (providing 32 and 8 channels, respectively) were adopted to increase the analog channels of the MCU to 40. Then, through a Bluetooth module (WH-BLE103), the collected data are further processed to the GUI of the system on the external devices with a screen (e.g., mobile phone and computer) wirelessly (Figure 1d). Similar to the sensing panel, a thin PI film was employed as a supporting material for the patterned layer of the metal electrode (Cu) that is an electrical pathway for all the electrical components. Thereafter, the aforementioned electronics were directly soldered on the PI layer printed with the conductive pattern accordingly. Furthermore, to tightly connect the circuits of the sensing and controlling units, two rectangular magnetics were placed at the ends of the panels. Finally, the substrate with the circuit was encapsulated between two PDMS layers for protection and skin attachment (Figures S1 and S2). The adhesive properties of the PDMS layers associated with skin interfacing behaviors could be adjusted by the mixing ratio of PDMS to curing agent; thus, the WH-sensor could be mounted on human skin tightly under movement without failure during its wireless operation (Figure 1e). Then, to further prove its flexibility and robust durability, the device was tested under various angles which were bending in both directions (inward and outward) and twisting to mimic realistic body motions (Figure 1f). Through these experimental studies, the robust stability of the WH-sensor as a wearable device was demonstrated.

**Figure 2:**
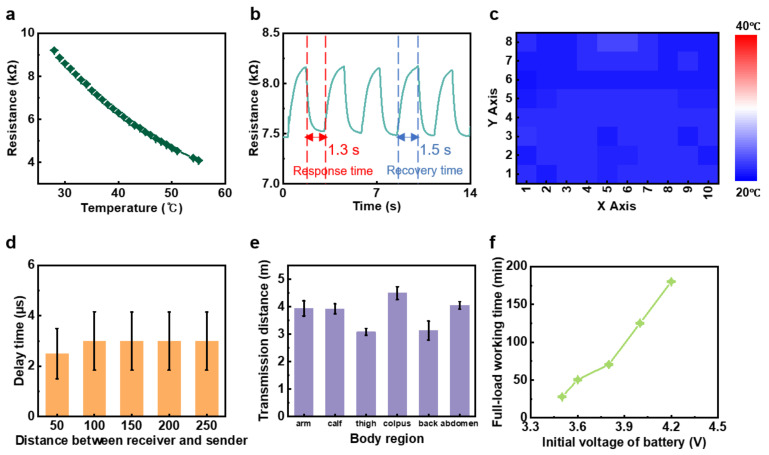
Electrical and operational performance of the WH-sensor. (**a**) Electrical signal of the thermistor versus temperature. (**b**) Electrical response of the NTC thermal sensor under controlled temperature (from 32 to 34 °C) (**c**) Temperature map of the WH-sensor at room temperature (21.5 °C) (**d**) Delay time of the device under distance variances. (**e**) Longest transmission distance at different body parts. (**f**) Full-load working duration of the device depending on the different initial voltages of the battery.

To investigate the sensitivity (beta (B) value) of the NTC thermistor whose resistance is inversely proportional to temperature, a range of heat (from 28 to 56 °C) was applied directly to the thermistor while its resistance was monitored at the same time (Figure 2a). The thermal resistor with 10 kΩ of resistance at 25 °C exhibited approximately 9.2 and 3.3 kΩ, respectively, at the minimum and maximum values of the range. Based on the measured parameters, approximately 3648 of the B values could be calculated back. In addition, the difference between the numbers is greatly distinct and this demonstrates the performance of the thermistor that is the major component for the thermal sensors. To further evaluate the sensitivity, the response and recovery time of the thermal resistor were examined by adopting two fixed temperatures (32 and 34 °C) repeatedly (Figure 2b). According to the schematic graph, it is clear that 1.3 s had been consumed to reach 7.5 kΩ, indicating 34 °C of heat applied from 8.2 kΩ (corresponding temperature of 32 °C), while it took 1.5 s for the recovery duration. Therefore, it was verified that the device possesses noticeable sensitivity due to its rapid transition duration. Subsequently, the GUI software developed for displaying the thermal levels of each subdivision of the target in colors (deep blue at 20 °C and deep red at 40 °C) (Figure S3) had been evaluated at 21.5 °C to confirm the functionality of the program (Figure 2c). As the fixed temperature that was fairly close to the minimum value of the GUI (20 °C) was applied to the sensors, all 80 sensing parts in the 8 × 10 (row × column) array were illustrated in blues. As a result, the accuracy of the thermal sensors and the GUI had been proved due to their corresponding data presentation. Thereafter, the operational performance of the WH-sensor was assessed to demonstrate its stability under different conditions. At first, the delay times of the data transmission under the various distances (50, 100, 150, 200, and 250 cm respectively) between the Bluetooth module and the external device for the display of the GUI program were tested. As shown in Figure 2d, except for the delay time at the distance of 50 cm (2.5 µs), all the other distances recorded 3 µs of lagging period identically. Consequently, it could be stated that the distance between the receiver and sender is not a considerable factor influencing the operation of the device while proving its stability. Following the experiment, the maximum transmission lengths between the target body parts with attached sensors (the arm, calf, thigh, colpus, back, and abdomen) and the data receiving device were determined (Figure 2e). As a result, it exhibited 3.94, 3.92, 3.08, 4.49, 3.13, and 4.05 m respectively. According to the resulting measured distances, the range of the working transmission distance (from 3.13 to 4.49 m) was verified and at the same time, it was illustrated that the device is functional in most of the body regions (Figure S4). Finally, the continuous operating durations of the WH-sensor based on its initial voltages of the power source (3.5, 3.6, 3.8, 4, and 4.2 V) were checked in Figure 2f. Certainly, a battery with a larger quantity of initial voltage showed a relatively longer running time, as the device was operated for 27.75, 50.5, 70.5, 125.25, and 180 min respectively, which would be sufficient for hemodynamic monitoring. Through the performance tests, the high accuracy and sensitivity, robust stability, and durability of the WH-sensor were demonstrated while it apparently showed that it could be operated wirelessly at a distance.

**Figure 3:**
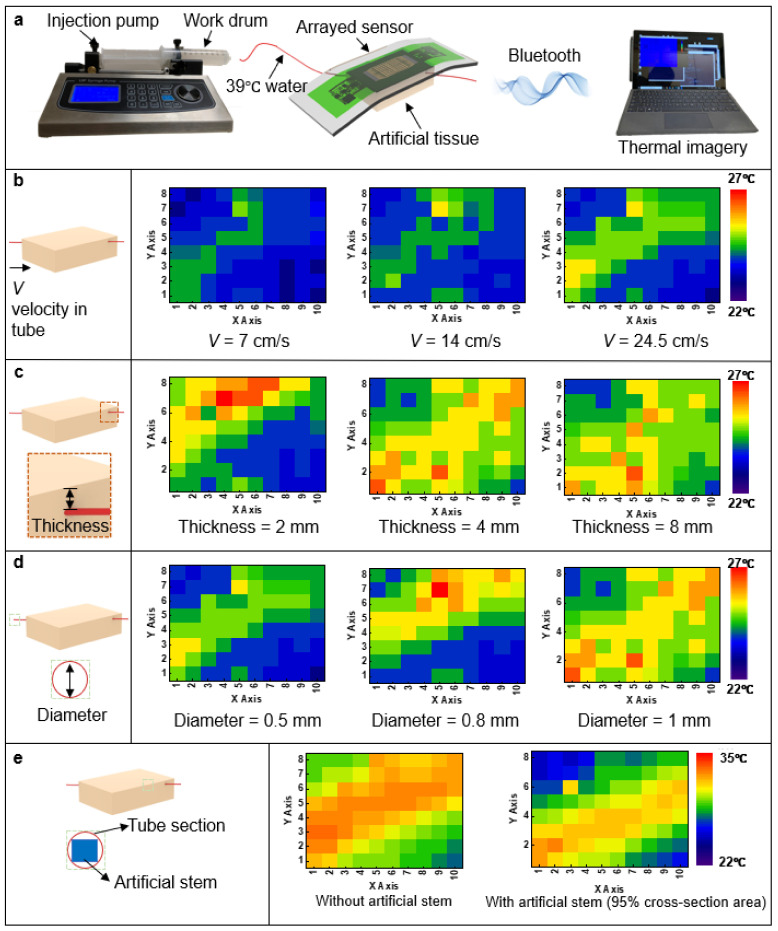
Experimental results of the WH-sensor. (**a**) Schematic illustration of the experimental setup of the device. Graphical presentation of the signal of the WH-sensor under controlled velocities of a fluid (**b**), depths of artificial tissue substrate (**c**), diameters of the artificial vessel (**d**), and without and with artificial stem blocked respectively during experiment (**e**).

Next, the hemodynamic monitoring capability of the WH-sensor had been examined through experiments under controlled conditions. Figure 3a shows the overall experimental setup with three major parts including a syringe pump, the WH-sensor attached to artificial tissue, and a computer for GUI. The pump mainly composed of an injection pump and a working drum controls the volumetric flow rate of a fluid by adjusting the pressure applied at the tip of it while the liquid streams through an artificial blood vessel (a rubber tube) at the other end. The flow of water, at 39 °C to imitate the characteristics of real blood, was manipulated by the syringe pump. Then, the arrays of thermal sensors of the WH-sensor measured the temperatures of each subdivision as the fluid passed through the artificial tissue (a mass of PDMS) embedded with the blood vessel. In addition, the sensing data were updated through the GUI program displayed on the screen of the computer wirelessly in real-time. All the variables for the experiments, including the velocity of the fluid, the depth and diameter of the embedded manufactured vessel, and the presence of an artificial stem that partially blocked the flow were kept constant with one being altered at a time (Figure 3b,e), as different blood vessels (i.e., arteries, veins, and capillaries) have particular values for those parameters. According to Figure 3b, tested with 7, 14, and 24.5 cm/s, respectively, it is highly distinct that when the velocity of the flowing substance increased, indicating higher blood pressure, it was represented by a higher temperature on the GUI of the device. This phenomenon is one of the features of heat dissipation: when there is a greater quantity of volumetric flow of a higher temperature medium, there are more chances for heat loss from the medium to the environment with relatively lower thermal energy. Furthermore, as shown in Figure 3c, the overall temperature sensed was reduced as the distance between the WH-sensor and the target blood vessel was extended (2, 4, and 8 mm); this condition is clearly clarified by Fourier’s Law of Heat Conduction [88]:

(1) $$\dot{Q}=-kA\frac{∆T}{∆x}$$

where $\dot{Q}$ is the rate of heat transfer, *k* is the thermal conductivity of the medium, *A* is the heat transfer area, ∆*T* is the temperature difference between two target points, and ∆*x* is the distance between the points, where the heat transfer rate is inversely proportional to the distance. Therefore, the depth of the blood vessel could be evaluated with the help of the thermal data as a larger depth would lead to a reduction of heat transfer. In addition, referring to Figure 3d, as the diameter of the artificial vessel increased (0.5, 0.8, and 1 mm), a higher thermal level was observed. Similar to the velocity of the flow, the increased diameter resulted in the expansion of the cross-sectional area of the vessel and a higher volumetric flow rate was obtained. Therefore, a higher range of heat could be observed as the diameter of the fabricated vessel was widened. To show the impact of the variables (the velocity, depth, and diameter) on the temperature mapping system, the percentages of pixels above certain temperatures, 24 °C for the velocity and diameter and 26 °C for the depth were calculated based on Figure 3b–d (Figure S5). Therefore, apparent percentage rises were observed for the velocity and diameter while a noticeable drop was detected for the depth. Lastly, the abnormal condition of blood flow blockage had been experimented with by inserting an artificial stem (PDMS block) into the manufactured vessel to imitate thrombus and cholesterol, which are major obstacles to blood circulation (Figure 3e). In this test, the stem covered approximately 95 % of the hole of the vessel and according to the GUI, a narrowed stream of heat was detected for the blocked one. Consequently, the presence of the obstacle in the blood vessel could be verified through the WH-sensor. As a result, hemodynamic status, the location and size of a blood vessel, and even the presence of blockage could be analyzed based on the thermal characteristics of the specific blood pathway that demonstrates the volumetric flow of the fluid and the rate of heat transfer.

**Figure 4:**
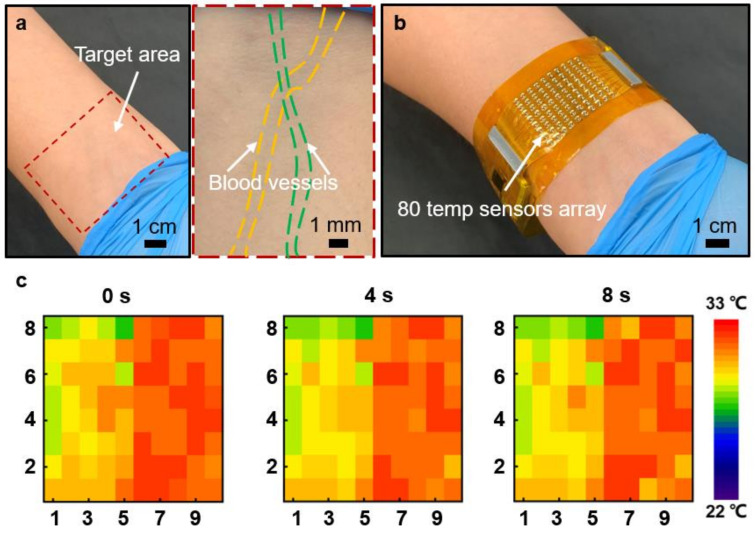
On-body real-time hemodynamic status monitoring. (**a**) Optical images of the target right wrist with the enlarged region showing the traces of the blood vessel. (**b**) Optical image of the WH-sensor attached to the target right wrist during operation. (**c**) Temperature map with the real-time measurements at 0, 4 and 8 s respectively.

After the experiments, a living-body test had been conducted to authenticate the performance of the hemodynamic status monitoring of the WH-sensor. As shown in Figure 4a, the right wrist was selected for the target as the locations of veins on the wrist are relatively apparent due to its thin layer of the epidermis. Therefore, the depth of the vein underneath the skin is superficial and is significant for thermal sensing. In addition, according to the enlarged optical image of Figure 4a, two target blood vessels (in this case, veins) could be clearly observed. For the real application experiment, the WH-sensor was directly attached to the target area for hemodynamic monitoring (Figure 4b). Especially, the sensing panel of the device installed with 80 thermal sensors was affixed at the target region as the sensing unit is the major component for sensing data, while the two controlling panels at the ends of the device process sensed data (Figure S6). Figure 4c exhibits the results of the on-body hemodynamic status monitoring at 0, 4, and 8 s in real-time. Through the measurements, an obvious color contrast had been observed, indicating that the left side of the target (bare skin without a blood vessel) possessed lower thermal levels, while the vein regions showed a higher temperature range. As a result, the location of the blood pathway could be detected precisely. Furthermore, the temperature of the bottom rows of the target was relatively higher than that of the top rows according to the GUI program. Therefore, the blood flow direction of the target vein could be analyzed, that is, from top to bottom (from hand to heart). In addition, unlike Figure 4a, although there are complicated blood vessel distributions underneath a target area for temperature mapping, the overall epidermal temperature would be significantly influenced by superficial veins due to their greater heat transfer rate. As a result, the device could perform successfully under different target areas. Consequently, the on-body experiment proved the functionality of the WH-sensor as a hemodynamic status monitoring device, including for blood vessel detection and its flow analysis.

## 3. Conclusions

In summary, the wearable and flexible hemodynamic monitoring sensor (WH-sensor) based on the thermal characteristics of the blood circulation system has been demonstrated. The combination of single sensing and two controlling panels provides 8 × 10 (row × column) of the NTC thermal sensors and simultaneously, the sensed data are wirelessly transmitted and displayed on the GUI. The temperature sensors proved their high sensitivity by showing the resistance change under a range of heat and instantaneous responses against the temperature changes. Furthermore, robust stability was presented by locating the device on the body parts at a distance during operation. Through the tests employing the artificial tissue, the blood pressure, vessel location, and a presence of blockage could be analyzed using different values of the parameters (the blood velocity, tissue depth, and vessel diameter). For the on-body experiment, the bare epidermis and the blood vessel could be exactly distinguished and even the flow direction could be detected at the same time. As a result, it could be stated that the performance of the device as a hemodynamic status monitoring sensor is significant, and it is highly convenient due to its simple operational steps. Therefore, it is expected that this device will be used as a POC device in the future.

## 4. Materials and Methods

### 4.1. Assembly of the WH Sensors

The fabrication firstly commenced with cleaning a quartz glass using acetone, alcohol, and deionized water (DI water) consecutively. A thin layer of polyimide (PI) with a thickness of 25 μm was attached to the glass sheet with double-sided tape. Following this, Au/Cr (200/40 nm) were deposited onto the PI film by magnetron sputtering using a machine (Q150TS, QUORUM) and then, through photolithography and etching, yielding metal was patterned in the desired geometries. For photolithography, a positive photoresist (PR, AZ 5214, AZ Electronic Materials) was spin-coated at 3000 rpm for 30 s, soft-baked on a hot plate at 110 °C for 4 min, and then exposed to ultraviolet light for 5 s. After that, the photoresist coated was developed by a developer (AZ 300MIF) for 15 s and then, by using acetone and DI water, the PR was rinsed away. In sequence, another layer of PI (2 μm, 3000 rpm for 30 s, annealed at 250 °C for 30 min) was spin-coated and to form insulation layers for the interconnected areas besides the regions of the electrodes, selectively etched by an Oxford Plasma-Therm 790 RIE system (patterns were defined by photolithography, similar to the previous step) at the power of 200 W for 10 min. Next, Au/Cr (200/40 nm) were sputtered onto the PI film, and then, through photolithography and etching, a conductive electrode for connecting sensors (thermistors) later was patterned in the desired position. After that, on the top layer of the sample, another layer of PR was spin-coated and underwent photolithography and etching to expose the bottom electrode to be connected to ACF later. Subsequently, by employing the silver paste, 80 thermistors were linked with the Au electrode. Finally, the whole sample was encapsulated by polydimethylsiloxane (PDMS, PDMS: crosslink = 30:1) via spin-coating and curing.

### 4.2. Assembly of the Sensing Panel for the WH Sensors

First of all, the fabrication commenced with cleaning a quartz glass using acetone, alcohol, and deionized water (DI water) consecutively. Sodium stearate aqueous solution as a thin sacrificial layer was spin-coated on the glass sheet and underwent drying process at 100 °C for 5 min. Next, a thin PDMS layer (0.17 mm) was spin-coated at 600 rpm for 30 s and baked at 110 °C for 5 min to form the flexible substrate. Before attaching the PI-supported circuit layer (Cu) to the cured PDMS layer, PDMS was spread to provide strong adhesion strength between the conductive film (Cu) and the substrate. Then, the layer of Cu film (6 µm) supported by the PI (12 µm) was flattened on the PDMS substrate and cured at 110 °C for 5 min. Next, through photolithography and etching, yielding metal was patterned in the desired configuration. During this procedure, for photolithography, a positive photoresist (PR, AZ 4620, AZ Electronic Materials) was spin-coated at 3000 rpm for 30 s, soft-baked on a hot plate at 110 °C for 5 min, and then, underwent ultraviolet (UV) light exposure for 45 s. After that, the PR coated device underwent development using a developer (AZ 400K) for 1 min and was etched by FeCl_2_ solution. By using acetone and DI water, unnecessary PR was rinsed away. Next, on the Cu/PI layer of the substrate, all the electrical components, including the microcontroller unit, multiplexers, Bluetooth module, resistors, capacitors, crystal oscillator, and bridge wires, were soldered according to its design using low-temperature solder joints. Finally, PDMS (145 kPa, 0.17 mm thick) was poured and cured at 110 °C for 5 min to encapsulate the device.

## Supplementary Materials

The following are available online at https://www.mdpi.com/article/10.3390/bios11110435/s1, Figure S1: The schematic illustration of the controlling panel of the WH-sensor, Figure S2: The schematic illustration of the circuit design of the controlling panel of the WH-sensor and its equivalent optical image, Figure S3: The GUI interface of the WH-sensor, Figure S4: Optical images of the WH-sensor attached to the abdomen, back, and calf during operation, Figure S5: (a) Percentage of pixels above 24 °C in Figure 3b. (b) Percentage of pixels above 26 °C in Figure 3c. (c) Percentage of pixels above 24 °C in Figure 3d, Figure S6: Optical images of the overall device and its single operating unit.

## References

[1] Kumar R., Rajasekaran M.P. An IoT based patient monitoring system using raspberry Pi. Proceedings of the 2016 International Conference on Computing Technologies and Intelligent Data Engineering (ICCTIDE’16).

[2] Zanjal S.V., Talmale G.R. (2016). Medicine reminder and monitoring system for secure health using IOT. Procedia Comput. Sci..

[3] Kaur P., Kumar R., Kumar M. (2019). A healthcare monitoring system using random forest and internet of things (IoT). Multimed. Tools Appl..

[4] Abawajy J.H., Hassan M.M. (2017). Federated internet of things and cloud computing pervasive patient health monitoring system. IEEE Commun. Mag..

[5] Hassanalieragh M., Page A., Soyata T., Sharma G., Aktas M., Mateos G., Kantarci B., Andreescu S. Health monitoring and management using Internet-of-Things (IoT) sensing with cloud-based processing: Opportunities and challenges. Proceedings of the 2015 IEEE International Conference on Services Computing.

[6] Wan J., Al-awlaqi M.A.A.H., Li M., O’Grady M., Gu X., Wang J., Cao N. (2018). Wearable IoT enabled real-time health monitoring system. EURASIP J. Wirel. Commun. Netw..

[7] Talal M., Zaidan A.A., Zaidan B.B., Albahri A.S., Alamoodi A.H., Albahri O.S., Alsalem M.A., Lim C.K., Tan K.L., Shir W.L. (2019). Smart home-based IoT for real-time and secure remote health monitoring of triage and priority system using body sensors: Multi-driven systematic review. J. Med. Syst..

[8] Ma Y., Choi J., Hourlier-Fargette A., Xue Y., Chung H.U., Lee J.Y., Wang X., Xie Z., Kang D., Wang H. (2018). Relation between blood pressure and pulse wave velocity for human arteries. Proc. Natl. Acad. Sci. USA.

[9] Li C., Han J., Ahn C.H. (2007). Flexible biosensors on spirally rolled micro tube for cardiovascular in vivo monitoring. Biosens. Bioelectron..

[10] Karpova E.V., Shcherbacheva E.V., Galushin A.A., Vokhmyanina D.V., Karyakina E.E., Karyakin A.A. (2019). Noninvasive diabetes monitoring through continuous analysis of sweat using flow-through glucose biosensor. Anal. Chem..

[11] Libby P., Buring J.E., Badimon L., Hansson G.K., Deanfield J., Bittencourt M.S., Tokgözoğlu L., Lewis E.F. (2019). Atherosclerosis. Nat. Rev. Dis. Primers.

[12] Madhvapathy S.R., Ma Y., Patel M., Krishnan S., Wei C., Li Y., Xu S., Feng X., Huang Y., Rogers J.A. (2018). Epidermal electronic systems for measuring the thermal properties of human skin at depths of up to several millimeters. Adv. Funct. Mater..

[13] Sherwani S.I., Khan H.A., Ekhzaimy A., Masood A., Sakharkar M.K. (2016). Significance of HbA1c test in diagnosis and prognosis of diabetic patients. Biomark. Insights.

[14] Cohn J.N., Finkelstein S., McVeigh G., Morgan D., LeMay L., Robinson J., Mock J. (1995). Noninvasive pulse wave analysis for the early detection of vascular disease. Hypertension.

[15] Strandness Jr D., Schultz R.D., Sumner D.S., Rushmer R.F. (1967). Ultrasonic flow detection: A useful technic in the evaluation of peripheral vascular disease. Am. J. Surg..

[16] Lau E.M., Manes A., Celermajer D.S., Galie N. (2011). Early detection of pulmonary vascular disease in pulmonary arterial hypertension: Time to move forward. Eur. Heart J..

[17] Bagavathiappan S., Saravanan T., Philip J., Jayakumar T., Raj B., Karunanithi R., Panicker T.M.R., Korath M.P., Jagadeesan K. (2009). Infrared thermal imaging for detection of peripheral vascular disorders. J. Med. Phys. Assoc. Med. Phys. India.

[18] Eun H.C. (1995). Evaluation of skin blood flow by laser Doppler flowmetry. Clin. Dermatol..

[19] Pugsley M., Tabrizchi R. (2000). The vascular system: An overview of structure and function. J. Pharmacol. Toxicol. Methods.

[20] Suzuki K. (2017). Overview of deep learning in medical imaging. Radiol. Phys. Technol..

[21] Antiga L., Ene-Iordache B., Remuzzi A. (2003). Computational geometry for patient-specific reconstruction and meshing of blood vessels from MR and CT angiography. IEEE Trans. Med. Imaging.

[22] Goo H.W., Goo J.M. (2017). Dual-energy CT: New horizon in medical imaging. Korean J. Radiol..

[23] Bateman T.M. (2012). Advantages and disadvantages of PET and SPECT in a busy clinical practice. J. Nucl. Cardiol..

[24] Spiegel P.K. (1995). The first clinical X-ray made in America—100 years. AJR Am. J. Roentgenol..

[25] Sivasubramanian M., Hsia Y., Lo L.-W. (2014). Nanoparticle-facilitated functional and molecular imaging for the early detection of cancer. Front. Mol. Biosci..

[26] Hendee W.R., O’Connor M.K. (2012). Radiation risks of medical imaging: Separating fact from fantasy. Radiology.

[27] Fazel R., Krumholz H.M., Wang Y., Ross J.S., Chen J., Ting H.H., Shah N.D., Nasir K., Einstein A.J., Nallamothu B.K. (2009). Exposure to low-dose ionizing radiation from medical imaging procedures. N. Engl. J. Med..

[28] Dorfman A.L., Fazel R., Einstein A.J., Applegate K.E., Krumholz H.M., Wang Y., Christodoulou E., Chen J., Sanchez R., Nallamothu B.K. (2011). Use of medical imaging procedures with ionizing radiation in children: A population-based study. Arch. Pediatrics Adolesc. Med..

[29] Hricak H., Brenner D.J., Adelstein S.J., Frush D.P., Hall E.J., Howell R.W., McCollough C.H., Mettler F.A., Pearce M.S., Suleiman O.H. (2011). Managing radiation use in medical imaging: A multifaceted challenge. Radiology.

[30] Mettler F.A., Mahesh M., Bhargavan-Chatfield M., Chambers C.E., Elee J.G., Frush D.P., Miller D.L., Royal H.D., Milano M.T., Spelic D.C. (2020). Patient exposure from radiologic and nuclear medicine procedures in the United States: Procedure volume and effective dose for the period 2006–2016. Radiology.

[31] Linet M.S., Slovis T.L., Miller D.L., Kleinerman R., Lee C., Rajaraman P., Berrington de Gonzalez A. (2012). Cancer risks associated with external radiation from diagnostic imaging procedures. CA Cancer J. Clin..

[32] Lin E.C. (2010). Radiation risk from medical imaging. Mayo Clin. Proc..

[33] Katholi R.E., Taylor G.J., McCann W.P., Woods W.T., Womack K.A., McCoy C.D., Katholi C.R., Moses H.W., Mishkel G.J., Lucore C.L. (1995). Nephrotoxicity from contrast media: Attenuation with theophylline. Radiology.

[34] Andreucci M., Solomon R., Tasanarong A. (2014). Side effects of radiographic contrast media: Pathogenesis, risk factors, and prevention. BioMed Res. Int..

[35] Beckett K.R., Moriarity A.K., Langer J.M. (2015). Safe use of contrast media: What the radiologist needs to know. Radiographics.

[36] Zhao Y., Kim A., Wan G., Tee B.C.K. (2019). Design and applications of stretchable and self-healable conductors for soft electronics. Nano Converg..

[37] Cheng I.-C., Wagner S. (2009). Overview of flexible electronics technology. Flexible Electronics.

[38] Nie B., Liu S., Qu Q., Zhang Y., Zhao M., Liu J. (2021). Bio-inspired Flexible Electronics for Smart E-skin. Acta Biomater..

[39] Nathan A., Ahnood A., Cole M.T., Lee S., Suzuki Y., Hiralal P., Bonaccorso F., Hasan T., Garcia-Gancedo L., Dyadyusha A. (2012). Flexible electronics: The next ubiquitous platform. Proc. IEEE.

[40] Gao W., Ota H., Kiriya D., Takei K., Javey A. (2019). Flexible electronics toward wearable sensing. Acc. Chem. Res..

[41] Nag A., Mukhopadhyay S.C., Kosel J. (2017). Wearable flexible sensors: A review. IEEE Sens. J..

[42] Yu X., Xie Z., Yu Y., Lee J., Vazquez-Guardado A., Luan H., Ruban J., Ning X., Akhtar A., Li D. (2019). Skin-integrated wireless haptic interfaces for virtual and augmented reality. Nature.

[43] Song E., Xie Z., Bai W., Luan H., Ji B., Ning X., Xia Y., Baek J.M., Lee Y., Avila R. (2021). Miniaturized electromechanical devices for the characterization of the biomechanics of deep tissue. Nat. Biomed. Eng..

[44] Liu Y., Zheng H., Zhao L., Liu S., Yao K., Li D., Yiu C., Gao S., Avila R., Chirarattananon P. (2020). Electronic skin from high-throughput fabrication of intrinsically stretchable lead zirconate titanate elastomer. Research.

[45] Wu M., Yao K., Li D., Huang X., Liu Y., Wang L., Song E., Yu J., Yu X. (2021). Self-Powered Skin Electronics for Energy Harvesting and Healthcare Monitoring. Mater. Today Energy.

[46] Wong T.H., Yiu C.K., Zhou J., Song Z., Liu Y., Zhao L., Yao K., Park W., Yoo W., Song E. (2021). Tattoo-like epidermal electronics as skin sensors for human-machine interfaces. Soft Sci..

[47] Han S., Kim J., Won S.M., Ma Y., Kang D., Xie Z., Lee K.-T., Chung H.U., Banks A., Min S. (2018). Battery-free, wireless sensors for full-body pressure and temperature mapping. Sci. Transl. Med..

[48] Crawford K.E., Ma Y., Krishnan S., Wei C., Capua D., Xue Y., Xu S., Xie Z., Won S.M., Tian L. (2018). Advanced approaches for quantitative characterization of thermal transport properties in soft materials using thin, conformable resistive sensors. Extrem. Mech. Lett..

[49] Gao Y., Yu L., Yeo J.C., Lim C.T. (2020). Flexible hybrid sensors for health monitoring: Materials and mechanisms to render wearability. Adv. Mater..

[50] Wang S., Chinnasamy T., Lifson M.A., Inci F., Demirci U. (2016). Flexible substrate-based devices for point-of-care diagnostics. Trends Biotechnol..

[51] Spanu A., Casula G., Cosseddu P., Lai S., Martines L., Pani D., Bonfiglio A. (2021). Flexible and wearable monitoring systems for biomedical applications in organic flexible electronics: Fundamentals, devices, and applications. Organic Flexible Electronics.

[52] Qaiser N., Al-Modaf F., Khan S.M., Shaikh S.F., El-Atab N., Hussain M.M. (2021). A Robust Wearable Point-of-Care CNT-Based Strain Sensor for Wirelessly Monitoring Throat-Related Illnesses. Adv. Funct. Mater..

[53] Ma Y., Zhang Y., Cai S., Han Z., Liu X., Wang F., Cao Y., Wang Z., Li H., Chen Y. (2020). Flexible hybrid electronics for digital healthcare. Adv. Mater..

[54] Yu X., Wang H., Ning X., Sun R., Albadawi H., Salomao M., Silva A.C., Yu Y., Tian L., Koh A. (2018). Needle-shaped ultrathin piezoelectric microsystem for guided tissue targeting via mechanical sensing. Nat. Biomed. Eng..

[55] Wang Y., Li K., Xu G., Chen C., Song G., Dong Z., Lin L., Wang Y., Xu Z., Yu M. (2021). Low-Cost and Scalable Platform with Multiplexed Microwell Array Biochip for Rapid Diagnosis of COVID-19. Research.

[56] Huang X., Li J., Liu Y., Wong T., Su J., Yao K., Zhou J., Huang Y., Li H., Li D. (2021). Epidermal self-powered sweat sensors for glucose and lactate monitoring. Bio-Des. Manuf..

[57] Wu M., Gao Z., Yao K., Hou S., Liu Y., Li D., He J., Huang X., Song E., Yu J. (2021). Thin, soft, skin-integrated foam-based triboelectric nanogenerators for tactile sensing and energy harvesting. Mater. Today Energy.

[58] Chen Y., Lu S., Zhang S., Li Y., Qu Z., Chen Y., Lu B., Wang X., Feng X. (2017). Skin-like biosensor system via electrochemical channels for noninvasive blood glucose monitoring. Sci. Adv..

[59] Michard F. (2016). Hemodynamic monitoring in the era of digital health. Ann. Intensive Care.

[60] Etemadi M., Inan O.T., Heller J.A., Hersek S., Klein L., Roy S. (2015). A wearable patch to enable long-term monitoring of environmental, activity and hemodynamics variables. IEEE Trans. Biomed. Circuits Syst..

[61] Wang Z., Wang S., Zeng J., Ren X., Chee A.J.Y., Yiu B.Y.S., Chung W.C., Yang Y., Yu A.C.H., Roberts R.C. (2016). High sensitivity, wearable, piezoresistive pressure sensors based on irregular microhump structures and its applications in body motion sensing. Small.

[62] Shaltis P., Reisner A., Asada H. Calibration of the photoplethysmogram to arterial blood pressure: Capabilities and limitations for continuous pressure monitoring. Proceedings of the 2005 IEEE Engineering in Medicine and Biology 27th Annual Conference.

[63] Yang T., Jiang X., Zhong Y., Zhao X., Lin S., Li J., Li X., Xu J., Li Z., Zhu H. (2017). A wearable and highly sensitive graphene strain sensor for precise home-based pulse wave monitoring. ACS Sens..

[64] Cheng W., Yu L., Kong D., Yu Z., Wang H., Ma Z., Wang Y., Wang J., Pan L., Shi Y. (2018). Fast-response and low-hysteresis flexible pressure sensor based on silicon nanowires. IEEE Electron Device Lett..

[65] Ryu S., Lee P., Chou J.B., Xu R., Zhao R., Hart A.J., Kim S.-G. (2015). Extremely elastic wearable carbon nanotube fiber strain sensor for monitoring of human motion. ACS Nano.

[66] Guo X., Huang Y., Zhao Y., Mao L., Gao L., Pan W., Zhang Y., Liu P. (2017). Highly stretchable strain sensor based on SWCNTs/CB synergistic conductive network for wearable human-activity monitoring and recognition. Smart Mater. Struct..

[67] Duan Z., Jiang Y., Huang Q., Yuan Z., Zhao Q., Wang S., Zhang Y., Tai H. (2021). A do-it-yourself approach to achieving a flexible pressure sensor using daily available materials. J. Mater. Chem. C.

[68] Dias D., Cunha J.P.S. (2018). Wearable health devices—Vital sign monitoring, systems and technologies. Sensors.

[69] Kim J., Chou E.-F., Le J., Wong S., Chu M., Khine M. (2019). Soft wearable pressure sensors for beat-to-beat blood pressure monitoring. Adv. Healthc. Mater..

[70] Bagavathiappan S., Saravanan T., Philip J., Jayakumar T., Raj B., Karunanithi R., Panicker T.M., Korath P., Jagadeesan K. (2008). Investigation of peripheral vascular disorders using thermal imaging. Br. J. Diabetes Vasc. Disease.

[71] Van de Staak W., Brakkee A., de Rijke-Herweijer H. (1968). Measurements of the thermal conductivity of the skin as an indication of skin blood flow. J. Investig. Dermatol..

[72] Sivakorn C., Schultz M.J., Dondorp A.M. (2021). How to monitor cardiovascular function in critical illness in resource-limited settings. Curr. Opin. Crit. Care.

[73] Shin J., Jeong B., Kim J., Nam V.B., Yoon Y., Jung J., Hong S., Lee H., Eom H., Yeo J. (2020). Sensitive wearable temperature sensor with seamless monolithic integration. Adv. Mater..

[74] Yang J., Wei D., Tang L., Song X., Luo W., Chu J., Gao T., Shi H., Du C. (2015). Wearable temperature sensor based on graphene nanowalls. RSC Adv..

[75] He Y., Li W., Han N., Wang J., Zhang X. (2019). Facile flexible reversible thermochromic membranes based on micro/nanoencapsulated phase change materials for wearable temperature sensor. Appl. Energy.

[76] Lin L., Chen Y.Y., Zhang X.X., Wang X.D. (2014). Optimization of geometry and flow rate distribution for double-layer microchannel heat sink. Int. J. Therm. Sci..

[77] Pottler K., Sippel C.M., Beck A., Fricke J. (1999). Optimized finned absorber geometries for solar air heating collectors. Sol. Energy.

[78] Rafati M., Hamidi A., Niaser M.S. (2012). Application of nanofluids in computer cooling systems (heat transfer performance of nanofluids). Appl. Therm. Eng..

[79] Jorfeldt L., Juhlin-Dannfelt A., Pernow B., Wassen E. (1978). Determination of human leg blood flow: A thermodilution technique based on femoral venous bolus injection. Clin. Sci. Mol. Med..

[80] Wei K., Jayaweera A.R., Firoozan S., Linka A., Skyba D.M., Kaul S. (1998). Basis for detection of stenosis using venous administration of microbubbles during myocardial contrast echocardiography: Bolus or continuous infusion?. J. Am. Coll. Cardiol..

[81] Hernandez-Divers S.M., Hernandez-Divers S.J., Wyneken J. (2002). Angiographic, anatomic and clinical technique descriptions of a subcarapacial venipuncture site for chelonians. J. Herpetol. Med. Surg..

[82] Pinsky M.R., Payen D. (2005). Functional hemodynamic monitoring. Crit. Care.

[83] Abraham W.T., Perl L. (2017). Implantable hemodynamic monitoring for heart failure patients. J. Am. Coll. Cardiol..

[84] Li D., Yao K., Gao Z., Liu Y., Yu X. (2021). Recent progress of skin-integrated electronics for intelligent sensing. Light Adv. Manuf..

[85] Liu Y., Zhao L., Avila R., Yiu C., Wong T., Chan Y., Yao K., Li D., Zhang Y., Li W. (2020). Epidermal electronics for respiration monitoring via thermo-sensitive measuring. Mater. Today Phys..

[86] Wang C., Cai M., Hao Z., Nie S., Liu C., Du H., Wang J., Chen W., Song J. (2021). Stretchable, multifunctional epidermal sensor patch for surface electromyography and strain measurements. Adv. Intell. Syst..

[87] Kim D.-H., Lu N., Ma R., Kim Y.-S., Kim R.-H., Wang S., Wu J., Won S.M., Tao H., Islam A. (2011). Epidermal electronics. Science.

[88] Liu I.-S. (1990). On Fourier’s law of heat conduction. Contin. Mech. Thermodyn..

