# Supplementary material for: High Channel Temperature Mapping Electronics in a Thin, Soft, Wireless Format for Non-Invasive Body Thermal Analysis
Source: Biosensors (Basel). 2021 Nov 2;11(11):435. doi: 10.3390/bios11110435 (PMC8615861; doi:10.3390/bios11110435)
Supplement: Supplementary file 1 [file biosensors-11-00435-s001.zip › biosensors-1415498-supplementary.pdf]

Supporting Information

High Channel Temperature Mapping Electronics in A Thin, Soft, Wireless Format for Non-invasive Body Thermal Analysis

Wooyoung Park [1,†], Chunki Yiu [1,2,†], Yiming Liu [1], Tsz Hung Wong [1], Xingcan Huang [1], Jingkun Zhou [1,2], Jian Li [1,2], Kuanming Yao [1], Ya Huang [1,2], Hu Li [1], Jiyu Li [1,2], Yanli Jiao [1], Rui Shi [1] and Xinge Yu [1,2,*]

[1] Department of Biomedical Engineering, City University of Hong Kong, Hong Kong 999077, China; wypark2@um.cityu.edu.hk (W.P.); chunkiyiu2-c@my.cityu.edu.hk (C.Y.); lyiming2-c@my.cityu.edu.hk (Y.L.); thwong247-c@my.cityu.edu.hk (T.H.W.); xhuang439-c@my.cityu.edu.hk (X.H.); jingkzhou3-c@my.cityu.edu.hk (J.Z.); jian.li@my.cityu.edu.hk (J.L.); km.Yao@my.cityu.edu.hk (K.Y.); yhuang@hkcoche.org (Y.H.); huli23@cityu.edu.hk (H.L.); jiyuli2-c@my.cityu.edu.hk (J.L.); yanlijiao2-c@my.cityu.edu.hk (Y.J.); rshi@connect.hku.hk (R.S.)

[2] Hong Kong Center for Cerebra-Cardiovascular Health Engineering, Hong Kong Science Park, New Territories, 999077 Hong Kong China

* Correspondence: xingeyu@cityu.edu.hk

† These authors contributed equally to this work.

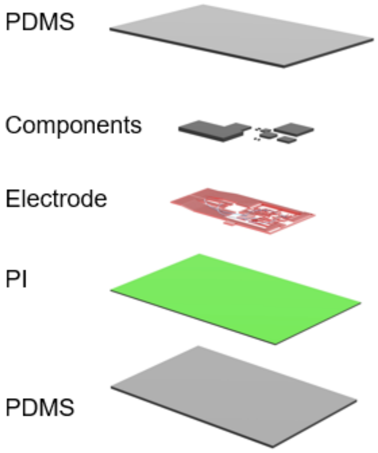

Figure S1. The schematic illustration of the controlling panel of the WH-sensor.

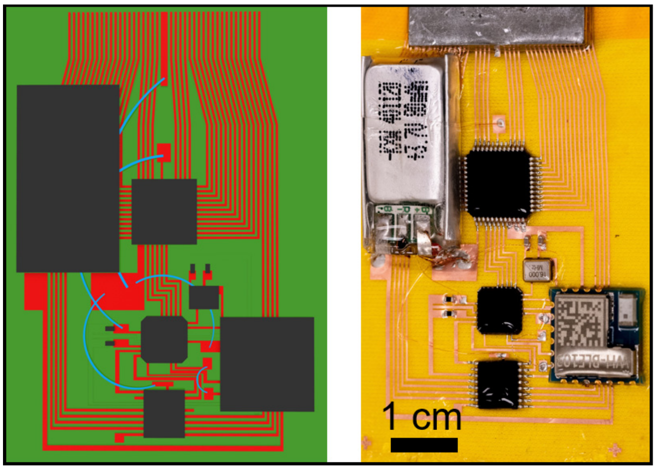

Figure S2. The schematic illustration of the circuit design of the controlling panel of the WH-sensor and its equivalent optical image.

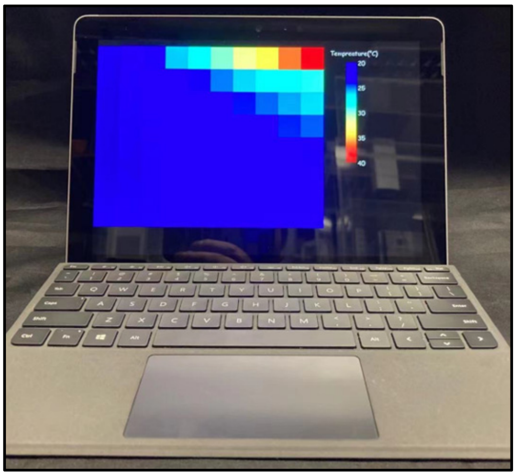

Figure S3. The GUI interface of the WH-sensor.

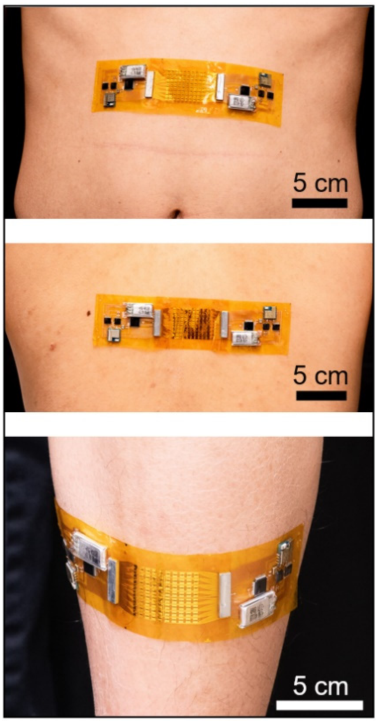

Figure S4. Optical images of the WH-sensor attached to the abdomen, back, and calf during operation.

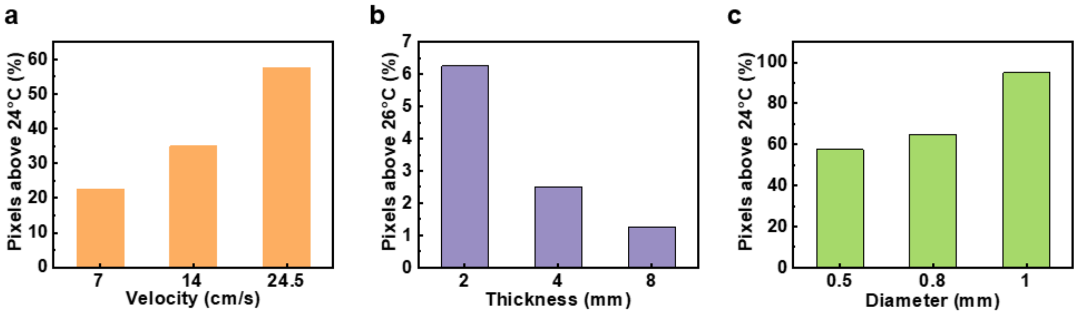

Figure S5. (a) Percentage of pixels above 24 °C in Figure 3b. (**b)** Percentage of pixels above 26 °C in **Fig. 3c**. **(c)** Percentage of pixels above 24 °C in Figure. 3d.

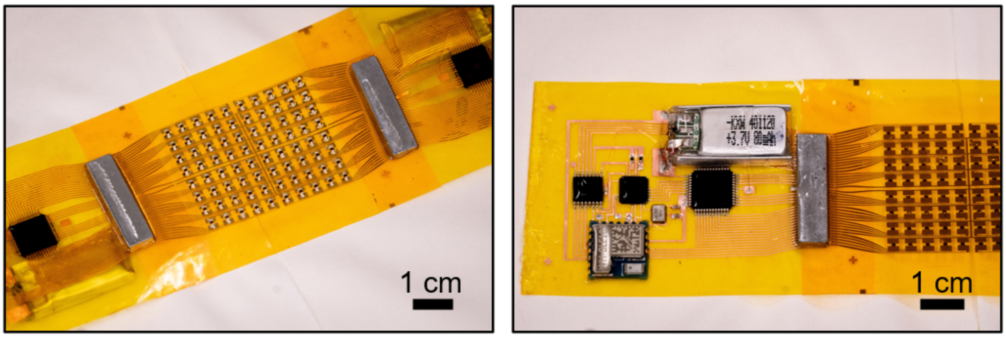

Figure S6. Optical images of the overall device and its single operating unit.
